# Large-Scale Genetic Structuring of a Widely Distributed Carnivore - The Eurasian Lynx (*Lynx lynx*)

**DOI:** 10.1371/journal.pone.0093675

**Published:** 2014-04-02

**Authors:** Eli K. Rueness, Sergei Naidenko, Pål Trosvik, Nils Chr. Stenseth

**Affiliations:** 1 Centre for Ecological and Evolutionary Synthesis (CEES), Dept. of Biosciences, University of Oslo, Oslo, Norway; 2 A. N. Severtsov Institute of Ecology and Evolution, Russian Academy of Sciences, Leninsky pr. 33, Moscow, Russia; Instituto de Higiene e Medicina Tropical, Portugal

## Abstract

Over the last decades the phylogeography and genetic structure of a multitude of species inhabiting Europe and North America have been described. The flora and fauna of the vast landmasses of north-eastern Eurasia are still largely unexplored in this respect. The Eurasian lynx is a large felid that is relatively abundant over much of the Russian sub-continent and the adjoining countries. Analyzing 148 museum specimens collected throughout its range over the last 150 years we have described the large-scale genetic structuring in this highly mobile species. We have investigated the spatial genetic patterns using mitochondrial DNA sequences (D-loop and cytochrome b) and 11 microsatellite loci, and describe three phylogenetic clades and a clear structuring along an east-west gradient. The most likely scenario is that the contemporary Eurasian lynx populations originated in central Asia and that parts of Europe were inhabited by lynx during the Pleistocene. After the Last Glacial Maximum (LGM) range expansions lead to colonization of north-western Siberia and Scandinavia from the Caucasus and north-eastern Siberia from a refugium further east. No evidence of a Berinigan refugium could be detected in our data. We observed restricted gene flow and suggest that future studies of the Eurasian lynx explore to what extent the contemporary population structure may be explained by ecological variables.

## Introduction

The population genetic structure of species of the northern Hemisphere has been shaped by a combination of historic and contemporary influences. The impact of the Pleistocene climatic oscillations on the biota has been thoroughly demonstrated (e.g. [1], [2]) and comparative studies of genetic variability in similarly distributed species have revealed common phylogeographic patterns, indicating the locations of glacial refugia and post-glacial colonization routes (e.g. [3], [4]).

It has been suggested that species with relatively high dispersal capacity and the ability to adapt to new habitats show less phylogeographic structuring than more stationary and specialized species. Examples of highly mobile terrestrial carnivore mammals that display little historical divergence on continental scales include the Canada lynx (*Lynx Canadensis* [5]), the bobcat (*Lynx rufus* [6]), the grey wolf (*Canis lupus* [7]) and the red fox (*Vulpes vulpes* [8]). On the small scale the same species may display non-random gene flow in the absence of geographical barriers. Such cryptic population structuring has been shown for instance in the Canada lynx [5], [9], the grey wolf [10], [11], the Scandinavian lynx (*Lynx lynx* [12]) and coyotes (*Canis latrans* [13]). It has been hypothesized that the differentiation observed in these studies has been caused by ecological factors. It was demonstrated through modelling that variable snow conditions (supposedly important for hunting capacity) could create differentiation in Canada lynx [14], [15], and more recently significant correlation between genetic distances and dietary differentiation was found for Eastern European grey wolves [16]. Altogether, there is growing evidence for associations among prey, habitat choice and population genetic structure in highly mobile carnivores.

Relatively few studies of widely distributed Eurasian species cover the extent of the Russian sub-continent. Nevertheless, a couple of remarkable general phylogeographical patterns have been observed and Korsten *et al.* [17] propose that the mitochondrial DNA genealogies of Eurasian mammals follow either of two general patterns:

1. Closely related mtDNA haplotypes are found throughout northern continental Eurasia, suggesting expansion of one maternal lineage (from a single refugium) into these areas (no significant dispersal barriers). Examples of this model include e.g. brown bear (*Ursus arctos* [17]), and a number of small mammals (see [18]).
2. Genetically distinct lineages meet in a suture zone near the Ural Mountains, which may represent a dispersal barrier. The Eurasian badger (*Meles meles*) and the tundra shrew (*Sorex tundrensis*) are examples where divergent phylogroups are separated by multiple substitutions [18], [19].

Here we provide the first description of the large-scale spatial genetic patterns of the Eurasian lynx throughout its entire range, the bulk (about 75%) of which lies within the borders of Russia [20], expanding into central Asia in the south and Europe in the west. The species is thus well suited to test the generality of the scenarios described above.

In the late Pleistocene, Eurasia and North America were connected by the Bering Land Bridge [21], and a common refugium, Beringia, existed during the last glacial maximum (LGM), around 20,000 years ago (e.g. [22]). The phylogenies of several mammalian species, or species pairs, with Holoarctic distributions have a Beringian clade containing individuals sampled both in Central Asia/eastern Siberia and North America (cf. [23], [24]). The Eurasian and Canadian lynx are believed to have diverged much earlier (1.6–1.2 million years ago, [25]) and their distributions are presumably non-overlapping. However, among the scarce fossil evidence that exists, one specimen classified as *Lynx lynx* has been found in North America [26] suggesting the possibility of a historical range overlap. Genetic data from lynx from the eastern part of Eurasia have not been analyzed previously.

Estimates of divergence times of monophyletic groups (based on molecular clock approaches) are commonly used to link past demographic expansions to paleoclimatic events. For several Eurasian species it has been demonstrated that the split between different genealogical lineages predates the LGM (see [27]). The timing of the Canadian/Eurasian lynx split (see above) is suitable as a calibration point for establishing a timeline for the phylogeographic events of the Eurasian lynx.The Eurasian lynx is one of the world’s most widespread feline species even though it was eradicated from many parts of it former range in Western Europe during the 20^th^ century. The quality of lynx habitats varies greatly over the Eurasian landmasses [28]. Most common are forested areas (coniferous, deciduous and mixed), but it also includes mountains (e.g. in China), and semi-desert (Kazakhstan). Lynxes are solitary predators that occur at low densities and forage over wide areas. Their diet consist mainly of small ungulates and hares, but they also prey on birds and rodents [28]. Adult animals occupy territories of varying sizes (e.g.[29]–[31]) and dispersal distances have been measured to up to 148 km [32].

In spite of this great niche diversity, by far most of the available knowledge on both ecology (e.g. spatial distribution, home range size, dispersion [29]–[31], [33]), and genetics [12], [34] is based upon data from the European part of the distribution area. The highest diversity has so far been described by Ratkiewicz *et al.* [35] who analysed a part of the mitochondrial DNA (mtDNA) control region of 190 lynx samples from Scandinavia, the Baltic States and Poland.

**Figure 1:**
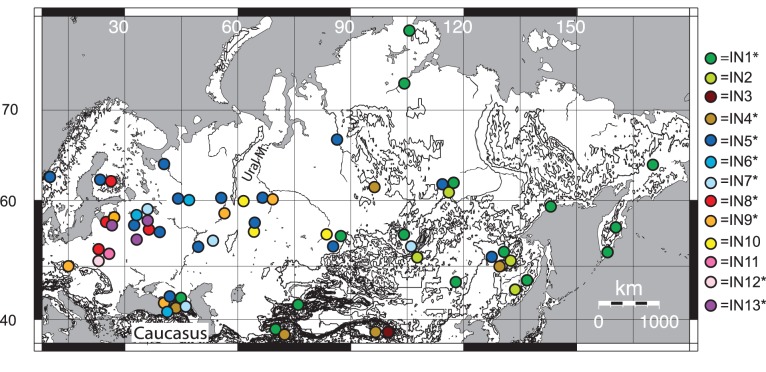
Map of the sampling area with geographic distribution of the haplotypes IN1–IN13 indicated by color-filled circles. The symbol * is used to indicate haplotypes that have been observed in other studies of Eurasian lynx by Hellborg *et al.* [34] and Gugolz *et al.* [59] in the following geographic areas: IN1 in Mongolia, Siberia and historical samples of Italian lynx, IN4 in Asia and Caucasus, IN5 in Scandinavia, Finland, the Baltics, Switzerland and historical samples of Italian lynx, IN8 in Finland, the Baltics and historical samples from Austria and Switzerland, IN9 in the Carpathian Mountains and IN13 in Finland and the Baltics.

We have analyzed samples covering the wide extent of Russia from Karelia to Kamchatka as well as adjoining areas like Belarus, Caucasus, China and Kazakhstan (Figure 1). Since the availability of samples of large and highly mobile solitary predators, like lynx, is very restricted we have utilized about 150 museum specimens collected between 1844 and 2002 (Table S1). We combine (mtDNA) sequences (*cytb* and D-loop) with microsatellite genotypes (11 loci) in order to assess the genetic variability of the Eurasian lynx throughout its range.

The specific aims of this study are: 1) to test whether or not the spatio-genetic variability of the Eurasian lynx throughout its distribution area follows any of the patterns described by Korsten *et al.* [17], 2) to estimate divergence times and shed new light on the historical, geographical and ecological factors underlying large-scale genetic structuring, 3) to investigate if genetic traces of a Beringian refugium may be detected. We present new information pertaining to each of these goals, describe restricted gene flow not caused by contemporary geographical barriers and outline some future perspectives.

## Materials and Methods

### Sampling, DNA Extraction and Amplification

The majority of the samples used in this study were taken from museum specimens mostly collected during the 1900 s (1844–2002). We obtained permission to access the collections and sample lynx skulls from the Zoological Museum of Moscow State University (Moscow), Zoological Museum of Zoological Institute (Saint-Petersburg), the Museum of Institute of Animal Systematics and Ecology (Novosibirsk) and received samples from Bergen Museum and Almaty Zoo. All of the specimens were donated by the museums/institutions. The samples were kept in separate paper envelopes until DNA extraction. Table S1 lists the source and reference number of each sample and contains information about quality and age of the samples, and geographical origin of the sampled individuals (only the samples that amplified successfully are included in this table, see below).

Bone powder was obtained by drilling into the cavities of craniums or the marrow of teeth, using a Dremmel 10.8 V Lithium_ion. All equipment, like gloves, drill bits and plastic bags used to cover the drill, was changed between handling each specimen in order to avoid contamination. We used the DNeasy Blood & Tissue Kit (QIAGEN), with a slightly modified protocol; the volume of ATL was increased to 300 μl, 200 μl EDTA (10 mM) and 20 μl proteinase K was added per reaction in the first step and the lysates were incubated on a shaker at 55°C for 24 h. An additional 10 μl of proteinase K were added after approximately 12 h. The volumes of AL and EtOH in step two were increased to 450 μl. We included one negative control for every eight samples. The yield of DNA was low and we did not attempt to quantify the exact amount for most of the samples.

Two fragments of mtDNA were amplified: 725 bp of the control region using the primers mtU and R3 [9], and 376 bp of *cytb* using the primers cytb1 and cytb 2 [36]. PCR reaction volumes were 20 μl with 1.125 U of HotStar *Taq* polymerase (QIAGEN), 2.5 μl HotStar PCR buffer, 2.5 nmol dNTP, 50 nmol MgCl2, 0.01 mg BSA and 8 pmol of each primer. The template DNA was vortexed for 10 seconds before it was added to the PCR mix. For samples that did not amplify the reaction volume was increased to 50 μl. The PCR was initiated by 15 min of denaturation at 95°C followed by 45 cycles of 30 sec at 94°C, 30 sec at 55°C and 1 min at 72°C. The program was terminated by 10 min at 72°C. Negative controls were always used. PCR products were treated with ExoSAP-IT (USB) prior to sequencing and electrophoresis on an ABI 3730 PRISM machine (Applied Biosystems).

The following eleven microsatellite loci were amplified: Fca001, Fca008, Fca031, Fca043, Fca045, Fca149, Fca 391, Fca506, Fca559, Fca628 and F115. A twelfth locus Fca441 was excluded due to low amplification rates. Primers for all of these loci have been developed for the domestic cat and have been mapped to different chromosomes [37]. The reaction mix per sample (10 μl each) contained: 0.25 U of HotStar *Taq* polymerase (QIAGEN), 1 μl HotStart PCR buffer (QIAGEN), 1 nmol dNTP, 10 nmol MgCl2, 2.5 pmol of each primer and template DNA. The PCR was initiated by 15 min of denaturation at 95°C followed by 40 cycles of 30 sec at 94°C, 45 sec at 52°C and 45 s min at 72°C. The program was terminated by 10 min at 72°C. We did not multiplex primers in the amplification reactions, but PCR products for 3–4 primers were pooled and a total volume of 1 μl was used for genotyping with an ABI PRISM 3730 apparatus. Ideally a multiple tube approach (e.g. [38]) should have been followed for all of the genotypes, unfortunately the amount of DNA available for some specimens was too limited. Routinely, a few samples were duplicated on each plate to check for consistency of the resulting genotypes.

### Amplification Success and Potential Errors

The amounts of DNA extracted from the museum specimens were generally very low and of the 200 samples we attempted to analyse 55 (27.5%) were excluded because they amplified for none or just a subset of the genetic markers. The 145 individuals that amplified for mtDNA D-loop and/or all of the 11 microsatellite loci are given in Table S1. Genotyping errors, such as allelic dropout and false alleles, may occur with a particularly high frequency in museum samples (e.g. [39]). In order to estimate the amplification error rates, a subset of the samples (12) were amplified four times for each marker. The software GIMLET [40] was used to calculate the frequencies of allelic dropout and false alleles. Museum samples have also been shown to be prone to single nucleotide misincorporation due to DNA damage in the template (e.g. [41]). We re-sequenced (different PCR products) all of the unique D-loop haplotypes and always got consistent results. Moreover, we observed much lower variability in the *cytb* compared to the D-loop region, which speaks against misincorporations as a major source of errors as the two regions would be equally exposed to DNA damage.

### Analysis of Genetic Variability

The sequences were aligned using MUSCLE [42] as incorporated in *MEGA* version 5 [43] which was also used to make an alignment of haplotypes based on informative sites only (ignoring polymorphisms observed only in single individuals). The software Network (Fluxus Technology Ltd.) was used to construct a median joning (MJ) network [44] connecting the mtDNA haplotypes based on informative sites (informative haplotypes).

The microsatellite data were analysed with the GeneMapper software (Applied Biosytems). Diversity indices were calculated in Arlequin ver 3.11 [45] using standard settings (i.e. assuming the infinite allele model). Arlequin was also used to calculate the observed and expected heterozygosity for each locus and to run an exact test of Hardy Weinberg equilibrium (HWE [46]) using a Markov Chain with a forecasted chain length of 1000000, and 10000 dememorization steps. The same software was used to perform a test for pairwise linkage disequilibrium [47], [48], with 10000 permutations and 10 initial conditions.

### Analysis of Genetic Structuring

Correlations between genetic and geographic distances were investigated through Mantel testing [49] carried out using the statistical programming interface R [50] with the package ‘ape’. We used the function ‘test.mantel’ with 20,000 permutations. The mtDNA distance matrix was computed with the function ‘dist.dna’ from the same package, using the ‘JC69’ algorithm [51] with zero gamma correction. For the genotypic data a shared allele distance was calculated using the individual-to-individual genetic distance calculator: http://www2.biology.ualberta.ca/jbrzusto/sharedst.php. For individuals with known year of sampling (see Table S1) Mantel tests for correlation between genetic distances and pairwise differences in sampling age, and between geographic distances and pairwise differences in sampling age, were performed in order to determine whether or not the variation in sample age could be a confounding factor.

Population structure in the microsatellite multilocus genotypes was investigated using GENELAND ver 3.1.4 [52], a software that provides a Bayesian clustering method for the inference of the number of subpopulations (K), and the spatial distribution of subpopulations. All unknown parameters are inferred through MCMC computations. We initially ran the MCMA 10 times allowing K to vary between 1 and 10 (it is possible that a higher K would have a higher likelihood, but considering the restricted number of samples the maximum number of K was set to 10), with 200000 MCMC steps, and the uncertainty of the spatial coordinates set to 2° (this value was chosen to account for uncertainty associated with determining exact sampling localities). The number of subpopulations was chosen from the modal of K from the 10 runs and we ran the MCMC chain 20 times with K fixed to this number leaving the other parameters unchanged. For comparison we also ran the non-spatial model in GENELAND and the software STRUCTURE [53], that perform Bayesian clustering analysis based on multilocus genotypes without geographic data. We used the same range of K and number of iterations as for the spatial analysis (see above).

GENELAND and STRUCTURE assign individuals attempting to make groups in HWE. As our sampling is scattered over large areas and many of the individuals may not fall into such a group we also wanted to analyse the genotype data without making any population genetic assumptions. The allelic distribution among individuals was therefore explored using Multiple Correspondence Analysis (MCA). MCA is a type of factor analysis applied to multivariate categorical data. It is essentially a singular value decomposition of an indicator matrix (i.e. a binary coding of the original factors [54]). This analysis provides a projection of the data onto a lower dimension space. MCA was carried out as described by Nenadic and Greenacre [55].

### Phylogenetic Analysis and Estimation of Divergence Times

The *cytb* and CR sequences were aligned to the equivalent sequences for the Canadian lynx (accession numbers AY31948–AY319505, CR and AY319506–AY319512 cytb) as well as sequences of the two other species of the *Lynx* genus, the Iberian lynx (*Lynx pardinus*) CR: AJ456979, cytb: AY499323 and the bobcat (*Lynx rufus)* CR: GQ979707 cytb: AY499331. Three datasets were analyzed:

1. The cytb haplotypes (382 bp) including comparable sequences for Canadian lynx (7 haplotypes described in [9]), Iberian lynx and bobcat.
2. A 553 bp fragment of the CR-region for which comparable sequences exist for 13 Canadian lynx haplotypes as well as haplotypes of Iberian lynx and bobcat, and
3. All full-length (725 bp) CR haplotypes with the only Canadian lynx haplotype available for the same fragment as outgroup.

MODELTEST [56] was used to select the model of nucleotide substitution that best fitted the data. The optimal model chosen using Akaike’s information criterion (AIC) was found to be HKY+Γ for all of the three datasets. We used BEAST v 1.7.4 [57] to investigate the demographic history and phylogeny of the Eurasian lynx. The analyses were performed using the HKY model of nucleotide substitution and Gamma distributed site heterogeneity with base frequencies estimated from the data. The tree model was Bayesian skyline plot sampling with 10 intervals. The chain length for each run was 10^7^ with parameters sampled every 1000 steps. Tracer v 1.5 [58] was used for diagnosing the MCMC output of each run. The maximum clade credibility target tree was found using TreeAnnotator and the target tree visualized by FigTree v1.4.0 (http://tree.bio.ed.ac.uk/software/figtree/). Divergence times, time since most recent common ancestor (tmrca), for different splits in the phylogenetic trees were based on age calibration on priors from Johnson *et al.* [25]. The root prior (i.e. the split between bobcat and the other lynx species) was 1.61 (1.06,2.60) Myr. Approximated by lnN(0.4762, 0.2445) where logmean is ln(1.61). The logsd parameter was found by doing a stepwise local search with fixed mean in order to minimize the sum of squared differences between the estimated confidence limits (0.70,1.98) and the 95% quantiles of 1000 theoretical lognormal distributions in the optimal neighborhood. The resulting optimal distribution produces the same expectation and 95% confidence limits as those estimated by Johnson *et al.* (13). The internal node prior (i.e. the split between *L. lynx* and *L. canadensis*) was 1.18 (0.70,1.98) Myr. Approximated by lnN(0.1655, 0.2641) where logmean is ln(1.18).

## Results

### Amplification Success and Genetic Diversity

**Figure 2:**
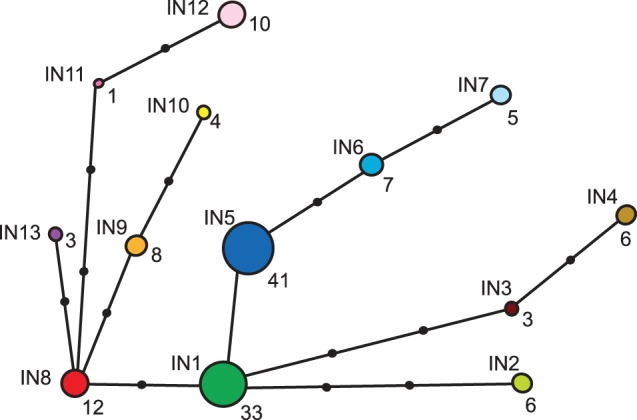
MJ-network of the 13 haplotypes defined by informative sites (IN1–IN13). The haplotypes are illustrated as colored circles and the sizes of the circles are proportional to the number of individuals with a given haplotype. The actual number of individuals is given as a bold number next to each haplotype. The black dots on the lines connecting the haplotypes symbolize point mutations.

Based on 725 bp of the D-loop control region (CR), from 137 individuals that amplified successfully, 48 unique haplotypes were defined by 68 nucleotide substitutions (three length mutations were not considered). As many as 37 of the haplotypes were singletons and the haplotype diversity was 0.90 (+/−0.02). The overall nucleotide diversity was 0.004 (+/−0.003) and the mean number of pairwise differences 3.23 (+/−1.68). Due to the high number of singletons we made a simplified alignment based on 15 informative sites that defined 13 CR haplotypes, IN1–IN13. A MJ network connecting the 13 haplotypes is presented in Figure 2. Little variability was observed for 382 bp of the *cytb* gene and we detected four haplotypes (designated A, B, C and D) defined by three point mutations in 56 sequenced individuals (haplotype diversity = 0.14+/−0.06). The most common haplotype was A (91%), three individuals displayed haplotype C while B and D was observed in one individual each, see Table S1.

A total of 104 samples amplified successfully for all of the 11 microsatellite loci. The number of alleles varied between five and 19 with a mean of 10. Table 1 lists the diversity indices as well as the error rates calculated for each locus. The overall amplification rate (4 replicates) was 0.86 (ranging from 0.58 to 1), the overall rate of allelic dropout was 0.018, with dropout occurring at four loci. The overall rate of false alleles was 0.018 and was only observed at one locus where two heterozygote individuals possessed genotypes from which only the larger allele amplified. The observed and expected heterozygosity for each locus is given in Table 1. Overall the observed heterozygosity (0.55) was lower than the expected heterozygosity (0.76). Nine of the eleven loci analyzed showed significant heterozygote deficiency. Linkage disequilibrium could not be detected between any pairs of loci.

**Table 1:** Variability of the microsatellite loci.

Locus	*n*	*ar*	*do*	*fa*	*Ho*	*He*	p
Fca001	17	0.94			0.68269	0.85586	>10^−5^
Fca008	7	0.98			0.65385	0.79241	0.1407
Fca031	10	0.79			0.49038	0.7841	>10^−5^
Fca043	8	0.92			0.56731	0.74684	>10^−5^
Fca045	5	0.69			0.47115	0.49814	0.61923
Fca149	6	1	0.050		0.35577	0.66843	>10^−5^
Fca391	9	0.92	0.056		0.33654	0.75766	>10^−5^
Fca506	10	0.58		0.20	0.48077	0.82256	>10^−5^
Fca559	9	1			0.65385	0.70778	0.02939
Fca628	10	0.83	0.095		0.69231	0.85763	0.00145
F115	19	0.77	0.023		0.69231	0.89929	>10^−5^
total	10	0.86	0.018	0.018			

n = number of alleles, ar = amplification rate, do = rate of allelic drop out, fa = rate of false alleles, Ho = observed heterozygosity, He = expected heterozygosity.

### Spatio-genetic Structuring

Figure 1 shows the geographical distribution of the sequenced individuals and the informative haplotype to which they assign. The most abundant haplotypes, IN1 (n = 33) and IN5 (n = 41), were widely distributed. IN8 and haplotypes derived from IN8 (IN9–IN13) were confined to the western part of the sampling area. IN5 is overlapping with both IN1 and IN8 on a west-east gradient, while IN1 and IN8 are non-overlapping. IN1–IN4 was detected only in the eastern and southern (Caucasus, Kazakhstan and China) parts of the sampling area.

Significant correlations between the genetic and geographical distances separating individuals were demonstrated both for the microsatellite, p<0.001, Figure S1 (A) and the D-loop data, p = 0.039, Figure S1 (B). The pairwise differences in sampling age were not significantly correlated to either genetic distances based on microsatellite data (C) or mitochondrial DNA data (D) or geographic distances (E). We therefore assume that the observed pattern of isolation by distance is not caused by the variability in sampling time.

**Figure 3:**
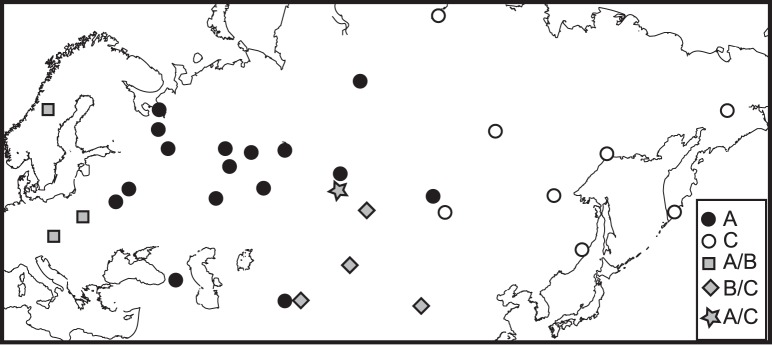
Assignment of individuals (based on their multilocus genotypes) to three clusters A (black dot), B and C (white dot), resulting from 20 runs in GENELAND, each point represents one or several individuals from the same location. No individuals were assigned to cluster B in all of the runs, but some were assigned alternately to A and B (square) or A and C (diamond). Two individuals assigned to A in some runs and C in others (star).

Ten runs of GENELAND with variable numbers of subpopulations (K) all suggested that the most likely alternative was that the data are structured into three subpopulations (K = 3). In 20 runs with K = 3 the majority of the individuals always assigned to one of two subpopulations defining western (A = 48%) and eastern (C = 23%) distributions (see Figure 3). A group of individuals (20%) sampled in the westernmost part of the study area (17 individuals sampled in the Białowieża Forest at the border between Poland and Belarus, two individuals sampled in the Carpatian Mountains in the Czheck Republic and two individuals sampled in Scandinavia in 1919) assigned to A in some runs and to the third subpopulation B in other runs. Some of the individuals (7%) sampled in the south (Kazaksthan and China) of the study area alternately assigned to subpopulations C and B. Western and southern lynx never assigned to cluster B in the same run. Only two individuals (2%) sampled in the middle of the study area (approximately 55°N 92°E) assigned to A in some runs and C in others. The non-spatial model in GENELAND resulted in K>3 showing that the clustering was affected by the geographic information. However, running STRUCTURE with K = 3, the percentage of individuals that assigned to clusters corresponding to those described above with a probability >0.90 was A = 27%, C = 23% and A/B = 16%, demonstrating that the genetic structuring defined by GENELAND did not solely rely on the geographic priors, see Figure S2.

### East-west Differentiation of Genotypes

**Figure 4:**
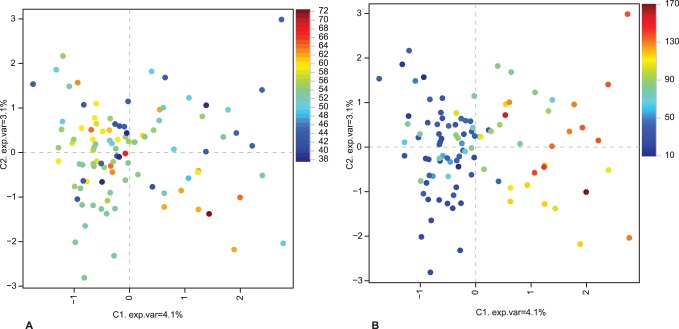
Allelic variation of 11 microsatellite loci displayed by the first and second variance components (PC1 and PC2). Each dot represents an individual and the colour-keys represent gradients in (A) latitude, and (B) longitude of the sampling locations.

Figure 4 displays the distribution of individuals as determined by the two primary variance components of the MCA model, 4.1% of the allelic variability was explained by the first variance component. The geographic distribution of the individuals, illustrated as a gradient of colours (from blue to red) reflecting increasing latitude (A) and longitude (B), clearly shows that the first variance component is related to the west-east axis of sampling sites. The range of longitude degrees (10–170) is much larger than that of latitude degrees (38–72), which corresponds to Euclidean distances of 3781 km and approximately 6000 km, respectively.

### Phylogeny and Divergence Times

**Figure 5:**
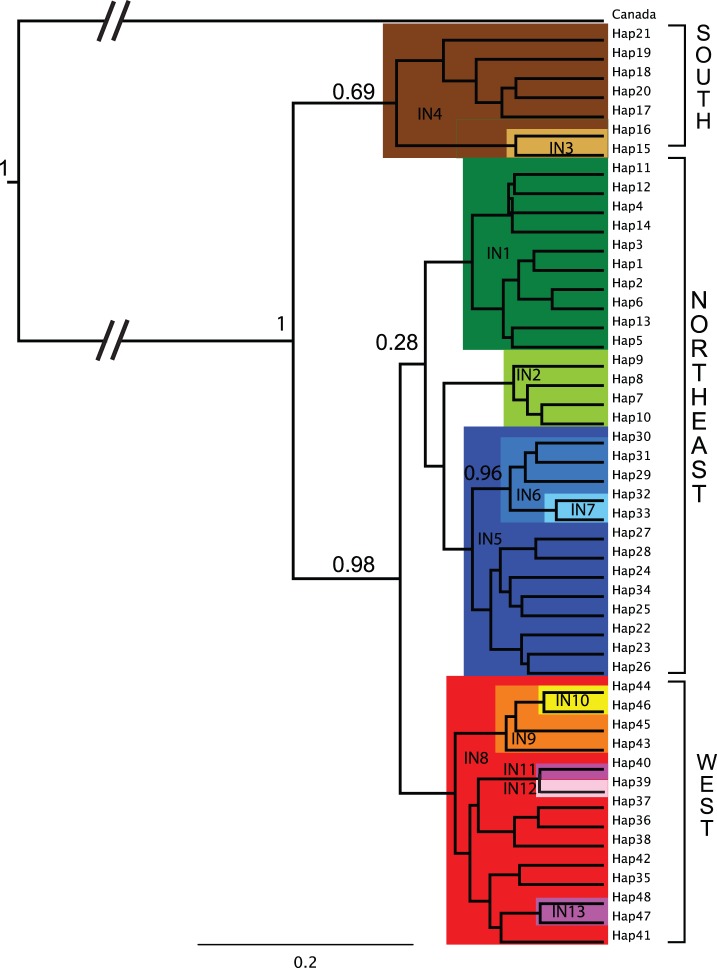
Phylogeny of the 48 CR haplotypes (725 bp). The 13 informative haplogroups, corresponding to IN1–IN13, are indicated by colours. Posterior probabilities are given next to some of the main branching points of the tree. We have defined three main clades, Clade South, Clade Northeast and Clade West. Canada lynx was used as outgroup.

The phylogeny (based on 725 bp of the CR) displaying the relationship among the 48 unique haplotypes (Figure 5) showed that three main clades have strong statistical support (posterior probabilities >0.98). Considering the geographic distribution of the haplotypes within each clade we name them West (red), Northeast (blue and green) and South (brown). Clade Northeast contains two main sub-clades (blue and green), but the spilt between them is not well supported (posterior probability 0.28). The haplotype diversity for each clade was: South = 0.92, West = 0.86 and Northeast = 0.80.

The divergence time estimates for each clade and for the Eurasian lynx (based on the three datasets) are listed in Table 2. The uncertainties around the time estimates are high, but it is suggested that most ancient lineages are found within clade South and that the Northeast clade predates clade West. A phylogeny based on 553 bp of the CR from Eurasian and Canadian lynx (Figure S3) shows that the two species are clearly separated into different clades.

**Table 2:** Estimated divergence times, in millon years.

tmrca (95% HPD)						
	root	split canadensis	Eurasia	Clade West	Clade South	Clade Northeast
Cytb	1.598 (2.239–0.970)	1.012 (1.426–0.604)	–	–	–	–
CR 553	1.693 (2.351–1.057)	0.944 (1.330–0.588)	0.237 (0.390–0.115)	0.0953 (0.139–0.041)	0.168(0.292–0.0639)	0.1211 (0.224–0.068)
CR 725	–	1.060 (1.624–0.560-	0.289 (0.499–0.087)	0.140 (0.242–0.050)	0.227 (0.433–0.057)	0.183 (0.315–0.070)

tmrca = time since most recent ancestor, HPD = highest posterior density.

## Discussion

Despite short genetic distances separating the main haplotypes, our results reveal a pronounced geographical structuring of the highly mobile Eurasian lynx. The most conspicuous pattern was genetic differentiation along a west-east axis and the same large-scale spatial pattern was observed for microsatellite and mtDNA data (Figure 1, Figure 3, Figure 4). We describe three main highly supported mtDNA clades West, Northeast and South (Figure 5) that partially overlap in their spatial distributions (Figure 1). All of the divergence estimates (Table 2) fall within the Pleistocene, which is typical for mammalian intra-specific phylogroups [2].

### Concurrence with Previous Genetic Studies of Eurasian Lynx

Nine of the 48 haplotypes (Table S1) have been described in previous studies of mtDNA diversity in Eurasian lynx from the westernmost part of the distribution range [34], [35], [59]. Their geographic distribution corresponds very well with our findings (see legend of Figure 1 for details). Altogether 12 haplotypes (H1–H12) have been defined earlier, and we find all of these with the exception of H7, H11 and H12. H7 was detected in two individuals sampled in Macedonia in south-eastern Europe [59] and would fall within our clade South. The most common haplotype of this clade, IN4, was detected (H5) in historic samples from Asia, Caucasus and Macedonia by Gugolz *et al.* [59]. Moreover, we did not detect haplotype H11 (differing from IN 13 by one length mutation) found in Polish lynx, and H12 (differing from IN5 by one substitution) found in Baltic lynx (cf. [35]). IN1 (H6) was detected in one historic sample (1824) of Italian lynx and in contemporary samples from Mongolia and Russia by Gugolz [59]. Ratkiewicz and co-authors [35] commented on the high haplotype diversity in the Baltic States, which can be explained by the suture zone between clade West and clade Northeast in this area.

### Glacial Refugia and Post-glacial Range Expansions

The geographical distributions of the three clades (Northeast, South and West) are only partially overlapping and a possible explanation for this is that populations of lynx may have been separated in different glacial refugia for shorter or longer periods resulting in genetic divergence. Alternatively, the clades could have diverged as a result of different post-glacial colonization routes followed by population expansions. During the Pleistocene, much of the lynx distribution range was alternately covered by glaciers during cooling periods and uncovered during warmer interglacials. All divergence estimates (Table 2) fall within the Pleistocene (1.8 mill to 10,000 years ago). Like for other mammals with wide Eurasian distributions the divergence times predate the LGM (e.g. [18] [17]). We will discuss possible scenarios for the shaping of the three clades separately.

#### The most ancient lynx lineages found in Central Asia

The south-eastern part of the lynx distribution range was not covered by extensive ice sheets during LGM and the vegetation was presumably not very different from today [60]. According to the estimates of divergence times (Table 2), the most ancestral group of lynx inhabits this area (clade South). It should be noted that the large uncertainty surrounding the estimates of divergence times calls for caution when interpreting these results. However, the highest level of haplotype diversity was observed within this clade, and the three rare *cytb* haplotypes were solely observed in clade South individuals. In order to investigate whether a similar pattern could be found for allelic diversity we counted the number of private alleles (found in one individual only) for the various geographical regions. Of the 18 alleles detected (in 104 individuals with complete multilocus genotypes), 9 were found among the 9 Lynx sampled in the South (of which 7 from China) while the rest were found in 69 individuals associated with the Northeastern clade. Altogether these results support the hypothesis of southern ancestry.

The Caucasus was the only sampling locality where haplotypes representing each of the three clades were detected. Caucasus is known as a biodiversity hotspot and the Caucasus forest refugium is the largest throughout the Western Asian/near Eastern region (e.g. [61]). Fossils of lynx have been found in cave sediments in the area [62]. The high haplotype diversity may, however, also result from secondary contact (i.e. post-glacial immigration from the North). Notably, only one individual sampled in the Caucasus represented the Western clade (haplotype IN9).

Altogether, our results strongly suggest that the present population of Eurasian lynx has had a longer history in the southern part of its range than elsewhere. A similar pattern with a distinct southeastern clade was observed in wood lemming (*Myopus schisticolor*) and several other boreal forest species (see [27]). For the grey wolf distinct genetic lineages are found in the Himalayas [63], a small population of Eurasian lynx occurs in the Himalayas [64] and should be analyzed in order to investigate if it represents ancient lineages.

#### Distinct European phylogroup

Several glacial refugia have been described in southern Europe (e.g. [4]) and more recently a smaller central/eastern European glacial refugium in the Carpathians (e.g. [65]). Fossil records confirm that Lynx were present in the Carpathians during LGM [66] and based on mtDNA data it has been suggested as a refugium for the species by Gugolz *et al.* [59] and Ratkiewicz *et al.* [35]. Our data from Carpathian lynx shows genetic homogeneity (all individuals display haplotype IN9) that could suggest past isolation. The genetic distance from other Western haplotypes is low, but the estimated node age of 0.095 (0,139–0.041) predates LGM. Haplotype IN9 and the derived IN10 were also found in several localities further east suggesting postglacial expansion.

The spatial distribution of the western clade is more restricted than that of the other two clades and all of the individuals analysed from Central and Eastern Europe assigned to this clade. The divergence time of the clade (Table 2) indicates that the uniqueness of the clade stems from historic isolation rather than postglacial colonization followed by restricted gene flow. Eurasian lynx fossils dated to the Pleistocene have also been found in southern Europe (e.g. [67]), but our data give no basis for locating other refugia.

#### Post-glacial expansion of the Northeastern clade

The Northwestern part of Russia and the Scandinavian Peninsula were completely covered by ice during the LGM (e.g. [68]). We find it very likely that the blue sub-clade (Figure 1 and Figure 5) diverged as a result of colonization of previously uninhabitable areas after the withdrawal of the LGM ice sheet. For instance, in Scandinavia only haplotype IN5 (hap22) was found, which was also the case for about 200 individuals sequenced by Hellborg *et al.* [34]. The three main haplotypes of the blue sub-clade (IN5, IN6, IN7) are all present in the Caucasus and we suggest the phylogroup originated in and spread from this area. The green sub-clade may have originated in a large boreal refugial area that existed in Eastern Siberia during the Late Pleistocene and the present distribution could result from demographic expansion into primarily the Northeastern parts of the lynx distribution range, similarly to what has been described for other boreal forest species (see [27]). The average number of private alleles per individual was considerably lower for the blue (0.02) than for the green (0.29) sub-clade. This finding is consistent with the scenario of differences in the demographic histories described above.

### No Traces of a Beringian Refugium

Phylogeographic studies of several mammals, both small rodents [69], [70] and large and highly mobile species like grey wolf [7], brown bear [71], red fox [72], moose (*Alces alces* [73]) and red deer (*Cervus elaphus* [74]) have revealed that North American and eastern Siberian/Asian haplotypes may form common clades, suggesting that these observations constitute the genetic remains of a Beringian refugium. Fossils of the extinct *Lynx issiodorensis*, the most likely progenitor to both Eurasian and Canadian lynx [22] show that this species was spread across both continents in the Early Pleistocene (*ca*. 1 Ma). Furthermore, fossils classified as *Lynx lynx* have been found in North America (the Paleobiology Database, http://paleodb.org). Nevertheless, a phylogenetic tree combining sequences of Eurasian and Canadian lynx showed that the two species are clearly separated and thus revealed no genetic remains of such a refugium in our data (Figure S3). This confirms that unlike for some other mammalian species-pairs (see [24]) the Bering Strait definitely separates these two species. For brown bear [71], [75], Beringian permafrost individuals have been analyzed, and revealed genetic diversity not detectable in the contemporary population. A similar approach should also be used for lynx in order to further investigate the possibility of an extinct Beringian population.

### Restricted Gene Flow

The geographical distribution of haplotypes (Figure 1) indicates that long distance dispersal does occur (some haplotypes are very widespread), but that gene flow is restricted (the distribution of the three main clades only partly overlap). Similarly, the non-random distribution of alleles, mainly along the west-east axis (Figure 3, Figure 4, Figure S2) indicates restricted gene flow. Historical discontinuity, postglacial colonization routes and subsequent suture zones may to a large degree explain the pattern of isolation by distance observed in the data. However, considering the long time passed since the withdrawal of the ice and the high dispersal capacity of lynx, additional factors must be considered.

The Ural Mountain range, sometimes considered a natural boundary between Europe and Asia, has been shown to hinder gene flow in small mammals like lemmings [70] and voles [69]. The east-west differentiation pattern observed in the genotype data (Figure 4) could be consistent with Ural as a dispersal barrier, but the presence of the same haplotypes on either side suggests that some gene flow takes place. However, this could result from dispersal around the southern part rather than across the Mountain Range. The species is known to inhabit the whole sub-alpine zone of higher mountains further south [28], [76], but we cannot conclude, based on our data, to what extent the Eurasian lynx is able to traverse the Ural Mountains.

Restricted gene flow in the absence of physical barriers over smaller geographic scales has been observed in lynx within Scandinavia [12] and in Poland [35], [77]. Behaviour ecological phenomena such as territoriality, natal philopatry and habitat preferences pertaining to hunting have been suggested as possible explanations [12]. Also for other wide ranging large carnivores restricted gene flow has been reported and sought explained by local adaptations to specific habitat or foraging conditions [72]. The possible ecological impact on gene flow patterns in lynx should be explored further on appropriate geographic scales, using high-resolution genetic and ecological data.

Male-biased dispersal is generally assumed to be common in mammals [78], also solitary ones [79]. On smaller spatial scales this phenomenon has also been described for bobcat [80] and Eurasian lynx [31], but not for the Canadian lynx [12], [81]. In species with pronounced female philopatry, stronger genetic differentiation is expected in maternally inherited mtDNA than in nuclear markers. We observed a significant effect of isolation by distance both in the mtDNA and microsatellite data (Figure S1 A and B) and there is no indication of male-biased dispersal of lynx on large geographic scales in our data. It should be noted that the correlation between genetic distances based on microsatellite data and sampling age (Figure S1, C) was marginally significant (p = 0.088) that could contribute to the effect of isolation by distance in the genotype data. There was also weak evidence for a positive correlation between sampling age and geographic distances (p = 0.142, Figure S1, E).

### Conclusions and Implications for Conservation

The large-scale phylogeographic pattern of the Eurasian lynx does not match perfectly with any of the two scenarios proposed by Korsten *et al.* [17]. Some haplotypes are found widespread throughout the northern continent (pattern 1), but there are also signs of long lasting sub-division. We were not able to detect any traces of a Beringian refugium. Gene flow is restricted (particularly on an west-east axis), but it is uncertain to what extent the Ural Mountains act as a dispersal barrier (pattern 2). A more fine-scaled sampling will be needed in order to study the population genetic structure in more detail, for instance to investigate if effects of population fragmentation caused by human activity may be detected in parts of the range. The effects of ecological variables that are known to vary over the distribution area of the Eurasian lynx, such habitat characteristics and diet [28], should be further investigated in order to explain the restricted gene flow. Moreover, application of modern high-throughput sequencing techniques could lead to a deeper understanding of the Eurasian lynx demographic history and local adaptations.

The Eurasian lynx has been divided into seven sub-species [20] based on geographical distribution of morphs, differing in traits like fur colour, body size and degree of spottedness. For instance the Caucasian subspecies (*Lynx lynx dinniki)* has several external characteristics such as small size and short, coarse fur with well-defined spots [28]. We observed no clear subdivision of the genetic data corresponding to these putative subspecies. The lack of genetic distinction between the subspecies could imply that to a large degree phenotypic traits like size, fur colour and fur texture are environmentally plastic. Considerable morphological differentiation (and division in subspecies), not reflected in genetic structuring occurs also for other carnivores such as brown bear, bobcat and grey wolf [6], [7], [17]. We propose that the three distinct Eurasian lynx clades described here are considered when defining units for conservation (evolutionary significant units [82]), in order to ensure that genetic variability will be maintained.

## Supporting Information

Figure S1 **Mantel tests for correspondence between genetic and geographical distances.** The panels show the observed Z-statistics (vertical lines) compared to the density distributions resulting from 20,000 permutations of the data matrices. (a) Microsatellite genetic distances against geographical distances and (b) mtDNA genetic distances against geographical distances, (c) sample age against microsatellite genetic distances, (d) sample age against mtDNA genetic distances and (e) sample age against geographic distances. The number of samples used for each test (n) and p-values (quantiles of the observed Z-statistics falling within the permutation distributions) are printed below each plot.
(EPS)

Figure S2 **Bar plot showing the assignment of individuals, based on multilocus genotypes, to each of three clusters (red, green, blue) corresponding to A, A/B and C in** **Figure 3** **.**
(EPS)

Figure S3 **Pylogenetic tree bases on 553 bp of the mtDNA control region showing that no clades are shared between Eurasian and North American lynx.** Posterior probabilities are given next to the branching points.
(EPS)

Table S1 **Sample information.** ID = identification number, for source abbreviations see below, reference number refers to that of the source collection, type is the quality of the sample, age is the year of sampling, CR = control region informative haplotype, CR* = control region haplotype, Acc. No = GenBank accession number, *cytb* = cytb haplotype, MS = microsatellite multilocus genotype.
(DOC)
