# Supplementary material for: Large-Scale Genetic Structuring of a Widely Distributed Carnivore - The Eurasian Lynx (Lynx lynx)
Source: PLoS One. 2014 Apr 2;9(4):e93675. doi: 10.1371/journal.pone.0093675 (PMC3973550; doi:10.1371/journal.pone.0093675)
Supplement: Table S1 — Sample information. ID = identification number, for source abbreviations see below, reference number refers to that of the source collection, type is the quality of the sample, age is the year of sampling, CR = control region informative haplotype, CR* = control region haplotype, Acc. No = GenBank accession number, cytb = cytb haplotype, MS = microsatellite multilocus genotype. (DOC) [file pone.0093675.s004.doc]

**Table S1. Sample information.** ID = identification number, for source abbreviations see below, reference number refers to

that of the source collection, Type is the quality of the sample, Age is the year of sampling, CR = control region informative

haplotype, CR* = control region haplotype, Acc. No = GenBank accession number, *cytb* = cytb haplotype,

MS = microsatellite multilocus genotype.

| # | ID | Source | Reference number | Type | Age | | Geographic coordinates  North East | | CR | | CR* | | Acc.no | | | *cytb* | | | Acc. no | | MS | | |  |
| --- | --- | --- | --- | --- | --- | --- | --- | --- | --- | --- | --- | --- | --- | --- | --- | --- | --- | --- | --- | --- | --- | --- | --- | --- |
| 1 | R3 | MM | S99069 | Bone | | 1974 | | | 60 | 24 | 44 | 15 | | --- | --- | --- | --- | | | IN6 | | Hap29 | | | EU818870 | | A | | | EU818890 | | | X | |
| 2 | R4 | MM | S103392 | Bone | | 1975 | | | 60 | 33 | 42 | 39 | | --- | --- | --- | --- | | | IN5 | | Hap22 | | |  | | A | | |  | | |  | |
| 3 | R6 | MM | S103393 | Bone | | 1975 | | | 60 | 33 | 42 | 39 | | --- | --- | --- | --- | | | IN5 | | Hap22 | | |  | | A | | |  | | |  | |
| 4 | R13 | MM | S82894 | Bone | | 1934 | | | 47 | 0 | 120 | 0 | | --- | --- | --- | --- | | | IN1 | | Hap14 | | | EU818855 | | A | | |  | | |  | |
| 5 | R15 | MM | S22352 | Bone | | 1936 | | | 43 | 35 | 40 | 42 | | --- | --- | --- | --- | | | IN4 | | Hap18 | | | EU818859 | | A | | |  | | |  | |
| 6 | R17 | MM | S22353 | Bone | | 1936 | | | 43 | 35 | 40 | 42 | | --- | --- | --- | --- | | | IN4 | | Hap17 | | |  | | C | | | EU818892 | | |  | |
| 7 | R21 | MM | S87934 | Bone | | 1968 | | | 62 | 26 | 117 | 32 | | --- | --- | --- | --- | | | IN5 | | Hap22 | | |  | |  | | |  | | |  | |
| 8 | R23 | MM | S100090 | Bone | | 1974 | | | 62 | 26 | 117 | 32 | | --- | --- | --- | --- | | | IN1 | | Hap1 | | |  | | A | | |  | | | X | |
| 9 | R24 | MM | S87935 | Bone | | 1968 | | | 62 | 26 | 117 | 32 | | --- | --- | --- | --- | | | IN2 | | Hap10 | | | EU818851 | | A | | |  | | |  | |
| 10 | R25 | MM | S100094 | Bone | | 1974 | | | 62 | 26 | 117 | 32 | | --- | --- | --- | --- | | | IN1 | | Hap1 | | |  | | A | | |  | | | X | |
| 11 | R26 | MM | S100093 | Bone | | 1974 | | | 62 | 26 | 117 | 32 | | --- | --- | --- | --- | | | IN1 | | Hap1 | | |  | | A | | |  | | | X | |
| 12 | R27 | MM | S95834 | Bone | | 1973 | | | 62 | 26 | 117 | 32 | | --- | --- | --- | --- | | | IN1 | | Hap1 | | |  | | A | | |  | | | X | |
| 13 | R28 | MM | S91750 | Bone | | 1971 | | | 62 | 26 | 117 | 32 | | --- | --- | --- | --- | | | IN1 | | Hap1 | | |  | |  | | |  | | |  | |
| 14 | R29 | MM | S100092 | Bone | | 1974 | | | 62 | 26 | 117 | 32 | | --- | --- | --- | --- | | | IN1 | | Hap1 | | |  | | A | | |  | | | X | |
| 15 | R30 | MM | S100091 | Bone | | 1974 | | | 62 | 26 | 117 | 32 | | --- | --- | --- | --- | | | IN1 | | Hap13 | | | EU818854 | |  | | |  | | | X | |
| 16 | R31 | MM | S92365 | Bone | | 1966 | | | 45 | 26 | 137 | 6 | | --- | --- | --- | --- | | | IN1 | | Hap2 | | | EU818843 | |  | | |  | | |  | |
| 17 | R32 | MM | S131518 | Bone | | NA | | | 50 | 13 | 130 | 14 | | --- | --- | --- | --- | | | IN4 | | Hap19 | | | EU818860 | | C | | |  | | | X | |
| 18 | R33 | MM | S101893 | Bone | | 1973 | | | 51 | 59 | 127 | 40 | | --- | --- | --- | --- | | | IN1 | | Hap12 | | | EU818853 | |  | | |  | | |  | |
| 19 | R36 | MM | S29926 | Bone | | 1936 | | | 45 | 3 | 136 | 36 | | --- | --- | --- | --- | | | IN2 | | Hap9 | | | EU818850 | | A | | |  | | | X | |
| 20 | R37 | MM | S112977 | Bone | | 1978 | | | 53 | 11 | 130 | 3 | | --- | --- | --- | --- | | | IN2 | | Hap8 | | | EU818849 | | A | | |  | | | X | |
| 21 | R38 | MM | S29167 | Bone | | 1932 | | | 49 | 31 | 139 | 41 | | --- | --- | --- | --- | | | IN1 | | Hap3 | | | EU818844 | |  | | |  | | | X | |
| 22 | R40 | MM | S91716 | Bone | | 1970 | | | 45 | 26 | 137 | 6 | | --- | --- | --- | --- | | | IN1 | | Hap1 | | |  | | A | | |  | | | X | |
| 23 | R41 | MM | S101892 | Bone | | 1973 | | | 51 | 59 | 127 | 40 | | --- | --- | --- | --- | | | IN5 | | Hap22 | | |  | | A | | |  | | | X | |
| 24 | R42 | MM | S29168 | Bone | | 1932 | | | 49 | 31 | 139 | 41 | | --- | --- | --- | --- | | | IN1 | | Hap1 | | | EU818842 | | A | | |  | | | X | |
| 25 | R43 | MM | S96813 | Bone | | NA | | | 45 | 55 | 133 | 44 | | --- | --- | --- | --- | | | IN2 | | Hap7 | | | EU818848 | | A | | |  | | |  | |
| 26 | R44 | MM | S41309 | Bone | | 1944 | | | 43 | 6 | 133 | 60 | | --- | --- | --- | --- | | | IN1 | | Hap1 | | |  | | A | | |  | | | X | |
| 27 | R45 | MM | S91715 | Bone | | 1970 | | | 45 | 26 | 137 | 6 | | --- | --- | --- | --- | | | IN1 | | Hap1 | | |  | | A | | |  | | | X | |
| 28 | R46 | MM | S131516 | Bone | | NA | | | 45 | 26 | 137 | 6 | | --- | --- | --- | --- | | | - | |  | | |  | | A | | |  | | | X | |
| 29 | R47 | MM | S131517 | Bone | | NA | | | 50 | 13 | 130 | 14 | | --- | --- | --- | --- | | | IN5 | | Hap22 | | |  | | A | | |  | | | X | |
| 30 | R49 | MM | S91714 | Bone | | 1970 | | | 45 | 26 | 137 | 6 | | --- | --- | --- | --- | | | IN1 | | Hap1 | | |  | | A | | |  | | |  | |
| 31 | R50 | MM | S49439 | Bone | | 1949 | | | 43 | 6 | 133 | 60 | | --- | --- | --- | --- | | | IN1 | | Hap11 | | | EU818852 | | A | | |  | | |  | |
| 32 | R51 | MM | S94178 | Bone | | 1965 | | | 43 | 0 | 76 | 0 | | --- | --- | --- | --- | | | IN1 | | Hap1 | | |  | | A | | |  | | |  | |
| 33 | R53 | MM | S40261 | Bone | | 1940 | | | 59 | 9 | 61 | 52 | | --- | --- | --- | --- | | | IN9 | | Hap45 | | | EU818886 | | A | | |  | | | X | |
| 34 | R54 | MM | S93084 | Bone | | NA | | | 55 | 56 | 64 | 26 | | --- | --- | --- | --- | | | IN5 | | Hap22 | | |  | | A | | |  | | | X | |
| 35 | R57 | MM | S14260 | Bone | | 1911 | | | 43 | 2 | 44 | 35 | | --- | --- | --- | --- | | | IN4 | | Hap17 | | | EU818858 | | C | | |  | | | X | |
| 36 | R58 | MM | S92919 | Bone | | NA | | | 55 | 56 | 64 | 26 | | --- | --- | --- | --- | | | IN10 | | Hap44 | | |  | | A | | |  | | |  | |
| 37 | R59 | MM | S81772 | Bone | | 1967 | | | 52 | 34 | 158 | 23 | | --- | --- | --- | --- | | | IN1 | | Hap4 | | | EU818845 | | A | | |  | | | X | |
| 38 | R60 | MM | S95916 | Bone | | 1973 | | | 64 | 39 | 170 | 24 | | --- | --- | --- | --- | | | IN1 | | Hap1 | | |  | |  | | |  | | | X | |
| 39 | R61 | MM | S17636 | Bone | | 1934 | | | 55 | 15 | 104 | 17 | | --- | --- | --- | --- | | | IN1 | | Hap1 | | |  | | A | | |  | | | X | |
| 40 | R62 | MM | S52259 | Bone | | 1951 | | | 53 | 0 | 23 | 0 | | --- | --- | --- | --- | | | IN8 | | Hap35 | | | EU818876 | | A | | |  | | | X | |
| 41 | R63 | MM | S55083 | Bone | | 1951 | | | 53 | 0 | 23 | 0 | | --- | --- | --- | --- | | | IN12 | | Hap39 | | | EU818880 | | A | | |  | | | X | |
| 42 | R64 | MM | S55089 | Bone | | 1952 | | | 53 | 0 | 23 | 0 | | --- | --- | --- | --- | | | IN12 | | Hap39 | | |  | | A | | |  | | | X | |
| 43 | R65 | MM | S36915 | Bone | | 1940 | | | 55 | 34 | 38 | 55 | | --- | --- | --- | --- | | | IN6 | | Hap30 | | | EU818871 | |  | | |  | | | X | |
| 44 | R66 | MM | S177898 | Bone | | 2002 | | | 56 | 55 | 32 | 46 | | --- | --- | --- | --- | | | IN5 | | Hap22 | | |  | | A | | |  | | | X | |
| 45 | R67 | MM | S14263 | Bone | | 1935 | | | 55 | 50 | 39 | 28 | | --- | --- | --- | --- | | | IN6 | | Hap29 | | |  | |  | | |  | | | X | |
| 46 | R68 | MM | S55082 | Bone | | 1951 | | | 53 | 0 | 23 | 0 | | --- | --- | --- | --- | | | IN12 | | Hap39 | | |  | | A | | |  | | | X | |
| 47 | R69 | MM | S55080 | Bone | | 1951 | | | 53 | 0 | 23 | 0 | | --- | --- | --- | --- | | | IN12 | | Hap39 | | |  | | A | | |  | | | X | |
| 48 | R70 | MM | S52260 | Bone | | 1951 | | | 53 | 0 | 23 | 0 | | --- | --- | --- | --- | | | IN12 | | Hap39 | | |  | | A | | |  | | | X | |
| 49 | R71 | MM | S55077 | Bone | | 1949 | | | 53 | 0 | 23 | 0 | | --- | --- | --- | --- | | | IN12 | | Hap39 | | |  | |  | | |  | | | X | |
| 50 | R72 | MM | S55075 | Bone | | 1948 | | | 53 | 0 | 23 | 0 | | --- | --- | --- | --- | | | IN8 | | Hap36 | | | EU818877 | | A | | |  | | | X | |
| 51 | R73 | MM | S55097 | Bone | | 1951 | | | 53 | 0 | 23 | 0 | | --- | --- | --- | --- | | | IN11 | | Hap40 | | | EU818881 | |  | | |  | | | X | |
| 52 | R74 | MM | S55087 | Bone | | 1951 | | | 53 | 0 | 23 | 0 | | --- | --- | --- | --- | | | IN8 | | Hap37 | | | EU818878 | |  | | |  | | | X | |
| 53 | R75 | MM | S40435 | Bone | | 1934 | | | 43 | 1 | 44 | 42 | | --- | --- | --- | --- | | | IN1 | | Hap5 | | | EU818846 | |  | | |  | | | X | |
| 54 | R76 | MM | S40435 | Bone | | 1932 | | | 64 | 5 | 40 | 38 | | --- | --- | --- | --- | | | IN5 | | Hap22 | | |  | | A | | |  | | | X | |
| 55 | R78 | MM | S76622 | Bone | | 1945 | | | 64 | 13 | 41 | 39 | | --- | --- | --- | --- | | | IN5 | | Hap22 | | |  | |  | | |  | | | X | |
| 56 | R80 | MM | S79792 | Bone | | 1967 | | | 55 | 50 | 39 | 28 | | --- | --- | --- | --- | | | IN8 | | Hap41 | | | EU818882 | |  | | |  | | | X | |
| 57 | R81 | MM | S42189 | Bone | | 1941 | | | 60 | 15 | 69 | 21 | | --- | --- | --- | --- | | | IN5 | | Hap24 | | | EU818865 | |  | | |  | | |  | |
| 58 | R82 | MM | S111101 | Bone | | 1977 | | | 55 | 59 | 92 | 48 | | --- | --- | --- | --- | | | IN10 | | Hap44 | | | EU818885 | |  | | |  | | | X | |
| 59 | R83 | MM | S92832 | Bone | | 1971 | | | 56 | 21 | 160 | 40 | | --- | --- | --- | --- | | | IN1 | | Hap1 | | |  | |  | | |  | | |  | |
| 60 | R84 | MM | S134442 | Bone | | 1977 | | | 55 | 59 | 92 | 48 | | --- | --- | --- | --- | | | IN5 | | Hap34 | | | EU818875 | |  | | |  | | | X | |
| 61 | R85 | MM | S94393 | Bone | | 1972 | | | 52 | 13 | 107 | 42 | | --- | --- | --- | --- | | | IN1 | | Hap1 | | |  | |  | | |  | | | X | |
| 62 | R86 | MM | S92255 | Bone | | 1971 | | | 52 | 13 | 107 | 42 | | --- | --- | --- | --- | | | IN2 | | Hap7 | | |  | | A | | |  | | | X | |
| 63 | R87 | MM | S55079 | Bone | | 1950 | | | 53 | 0 | 23 | 0 | | --- | --- | --- | --- | | | IN8 | | Hap36 | | |  | |  | | |  | | | X | |
| 64 | R88 | MM | S55084 | Bone | | 1951 | | | 53 | 0 | 23 | 0 | | --- | --- | --- | --- | | | IN8 | | Hap35 | | |  | |  | | |  | | | X | |
| 65 | R89 | MM | S55076 | Bone | | 1948 | | | 53 | 0 | 23 | 0 | | --- | --- | --- | --- | | | IN8 | | Hap38 | | | EU818879 | |  | | |  | | | X | |
| 66 | R90 | MM | S55081 | Bone | | 1951 | | | 53 | 0 | 23 | 0 | | --- | --- | --- | --- | | | IN12 | | Hap39 | | |  | |  | | |  | | | X | |
| 67 | R91 | MM | S55088 | Bone | | 1952 | | | 53 | 0 | 23 | 0 | | --- | --- | --- | --- | | | IN12 | | Hap39 | | |  | |  | | |  | | | X | |
| 68 | R92 | MM | S55085 | Bone | | 1951 | | | 53 | 0 | 23 | 0 | | --- | --- | --- | --- | | | IN12 | | Hap39 | | |  | |  | | |  | | | X | |
| 69 | R93 | MM | S55086 | Bone | | 1951 | | | 53 | 0 | 23 | 0 | | --- | --- | --- | --- | | | IN12 | | Hap39 | | |  | |  | | |  | | | X | |
| 70 | R94 | MM | S14195 | Bone | | 1926 | | | 60 | 34 | 55 | 58 | | --- | --- | --- | --- | | | IN5 | | Hap25 | | | EU818866 | |  | | |  | | | X | |
| 71 | R95 | MM | S106524 | Bone | | 1976 | | | 55 | 9 | 36 | 34 | | --- | --- | --- | --- | | | IN13 | | Hap48 | | | EU818889 | |  | | |  | | | X | |
| 72 | R96 | MM | S3073 | Bone | | 1910 | | | 59 | 5 | 30 | 9 | | --- | --- | --- | --- | | | IN5 | | Hap26 | | | EU818867 | |  | | |  | | | X | |
| 73 | R97 | MM | S14233 | Bone | | 1993 | | | 54 | 33 | 33 | 11 | | --- | --- | --- | --- | | | IN13 | | Hap47 | | | EU818888 | |  | | |  | | | X | |
| 74 | R98 | MM | S14193 | Bone | | 1935 | | | 55 | 50 | 39 | 28 | | --- | --- | --- | --- | | | IN6 | | Hap29 | | |  | |  | | |  | | | X | |
| 75 | R99 | MM | S2116 | Bone | | 1910 | | | 60 | 9 | 32 | 32 | | --- | --- | --- | --- | | | IN5 | | Hap23 | | | EU818864 | |  | | |  | | | X | |
| 76 | R100 | MM | S111100 | Bone | | 1977 | | | 58 | 23 | 56 | 48 | | --- | --- | --- | --- | | | IN9 | | Hap43 | | | EU818884 | |  | | |  | | |  | |
| 77 | R101 | MM | S6407 | Bone | | 1930 | | | 72 | 30 | 104 | 26 | | --- | --- | --- | --- | | | IN1 | | Hap1 | | |  | |  | | |  | | | X | |
| 78 | R103 | SPM | 15753 | Bone | | 1929 | | | 55 | 24 | 55 | 33 | | --- | --- | --- | --- | | | IN7 | | Hap32 | | | EU818873 | | A | | |  | | | X | |
| 79 | R104 | SPM | 14390 | Bone | | 1929 | | | 44 | 27 | 40 | 10 | | --- | --- | --- | --- | | | IN5 | | Hap22 | | | EU818863 | |  | | |  | | | X | |
| 80 | R105 | SPM | 14389 | Bone | | 1929 | | | 44 | 27 | 40 | 10 | | --- | --- | --- | --- | | | IN5 | | Hap22 | | |  | |  | | |  | | | X | |
| 81 | R106 | SPM | 14391 | Bone | | 1929 | | | 44 | 27 | 40 | 10 | | --- | --- | --- | --- | | | IN5 | | Hap22 | | |  | |  | | |  | | | X | |
| 82 | R107 | SPM | 14388 | Bone | | 1929 | | | 44 | 27 | 40 | 10 | | --- | --- | --- | --- | | | IN5 | | Hap22 | | |  | |  | | |  | | |  | |
| 83 | R108 | SPM | 14392 | Bone | | 1929 | | | 44 | 27 | 40 | 10 | | --- | --- | --- | --- | | | IN7 | | Hap32 | | |  | |  | | |  | | | X | |
| 84 | R111 | SPM | 1351 | Bone | | 1844 | | | 42 | 33 | 43 | 52 | | --- | --- | --- | --- | | | IN1 | | Hap1 | | |  | | A | | |  | | | X | |
| 85 | R112 | SPM | 14767 | Bone | | 1929 | | | 61 | 7 | 96 | 30 | | --- | --- | --- | --- | | | IN4 | | Hap17 | | |  | | A | | |  | | |  | |
| 86 | R116 | SPM | 10681 | Bone | | 1916 | | | 53 | 26 | 107 | 30 | | --- | --- | --- | --- | | | IN7 | | Hap32 | | |  | | A | | |  | | | X | |
| 87 | R118 | SPM | 35333 | Bone | | 1985 | | | 53 | 22 | 49 | 49 | | --- | --- | --- | --- | | | IN5 | | Hap22 | | |  | | A | | |  | | | X | |
| 88 | R120 | SPM | 9407 | Bone | | 1911 | | | 58 | 30 | 31 | 19 | | --- | --- | --- | --- | | | - | |  | | |  | |  | | |  | | | X | |
| 89 | R123 | SPM | 35362 | Bone | | 1961 | | | 59 | 13 | 33 | 34 | | --- | --- | --- | --- | | | - | |  | | |  | |  | | |  | | | X | |
| 90 | R124 | SPM | 35363 | Bone | | 1961 | | | 59 | 13 | 33 | 34 | | --- | --- | --- | --- | | | IN7 | | Hap33 | | | EU818874 | |  | | |  | | | X | |
| 91 | R125 | SPM | 31245 | Bone | | 1977 | | | 58 | 48 | 33 | 23 | | --- | --- | --- | --- | | | IN8 | | Hap35 | | |  | |  | | |  | | | X | |
| 92 | R126 | SPM | 15754 | Bone | | 1929 | | | 55 | 24 | 55 | 33 | | --- | --- | --- | --- | | | IN7 | | Hap32 | | |  | |  | | |  | | |  | |
| 93 | R127 | SPM | 14396 | Bone | | 1929 | | | 44 | 27 | 40 | 10 | | --- | --- | --- | --- | | | IN5 | | Hap22 | | |  | |  | | |  | | | X | |
| 94 | R128 | SPM | 14394 | Bone | | 1929 | | | 44 | 27 | 40 | 10 | | --- | --- | --- | --- | | | IN8 | | Hap42 | | | EU818883 | | A | | |  | | |  | |
| 95 | R129 | SPM | 14393 | Bone | | 1929 | | | 44 | 27 | 40 | 10 | | --- | --- | --- | --- | | | IN6 | | Hap29 | | |  | |  | | |  | | | X | |
| 96 | R131 | SPM | 35310 | Bone | | 1992 | | | 67 | 16 | 86 | 38 | | --- | --- | --- | --- | | | - | |  | | |  | |  | | |  | | | X | |
| 97 | R132 | SPM | 19599 | Bone | | 1932 | | | 41 | 50 | 43 | 22 | | --- | --- | --- | --- | | | - | |  | | |  | |  | | |  | | | X | |
| 98 | R134 | SPM | 23686 | Bone | | 1948 | | | 76 | 10 | 105 | 53 | | --- | --- | --- | --- | | | IN1 | | Hap1 | | |  | |  | | |  | | |  | |
| 99 | R135 | SPM | 34307 | Bone | | 1993 | | | 59 | 37 | 30 | 32 | | --- | --- | --- | --- | | | IN8 | | Hap35 | | |  | |  | | |  | | |  | |
| 100 | R139 | SPM | 15172 | Bone | | 1929 | | | 44 | 35 | 134 | 55 | | --- | --- | --- | --- | | | IN2 | | Hap7 | | |  | |  | | |  | | |  | |
| 101 | R141 | SPM | 18658 | Bone | | 1935 | | | 59 | 18 | 143 | 16 | | --- | --- | --- | --- | | | IN1 | | Hap6 | | | EU818847 | |  | | |  | | | X | |
| 102 | R143 | SPM | 19598 | Bone | | 1932 | | | 67 | 16 | 86 | 38 | | --- | --- | --- | --- | | | IN5 | | Hap22 | | |  | |  | | |  | | |  | |
| 103 | R146 | SPM | 35189 | Bone | | 2001 | | | 59 | 37 | 30 | 32 | | --- | --- | --- | --- | | | IN8 | | Hap35 | | |  | |  | | |  | | | X | |
| 104 | R147 | SPM | 21853 | Bone | | 1930 | | | 55 | 10 | 92 | 0 | | --- | --- | --- | --- | | | IN5 | | Hap22 | | |  | | A | | |  | | | X | |
| 105 | R148 | SPM | 29551 | Bone | | 1969 | | | 59 | 27 | 33 | 52 | | --- | --- | --- | --- | | | IN12 | | Hap39 | | |  | |  | | |  | | | X | |
| 106 | R152 | SPM | 19842 | Bone | | 1935 | | | 60 | 20 | 30 | 54 | | --- | --- | --- | --- | | | - | |  | | |  | |  | | |  | | | X | |
| 107 | R155 | SPM | 32152 | Bone | | 1969 | | | 51 | 36 | 87 | 39 | | --- | --- | --- | --- | | | - | |  | | |  | |  | | |  | | | X | |
| 108 | R158 | SPM | 26554 | Bone | | 1956 | | | 59 | 3 | 30 | 26 | | --- | --- | --- | --- | | | IN6 | | Hap31 | | | EU818872 | |  | | |  | | | X | |
| 109 | R161 | SPM | 32177 | Bone | | 1981 | | | 50 | 0 | 83 | 0 | | --- | --- | --- | --- | | | IN1 | | Hap1 | | |  | |  | | |  | | | X | |
| 110 | R162 | SPM | 32133 | Bone | | 1983 | | | 50 | 28 | 83 | 9 | | --- | --- | --- | --- | | | IN5 | | Hap22 | | |  | |  | | |  | | | X | |
| 111 | R163 | SPM | 32132 | Bone | | 1983 | | | 50 | 28 | 83 | 9 | | --- | --- | --- | --- | | | IN5 | | Hap22 | | |  | |  | | |  | | | X | |
| 112 | R165 | SPM | 1325 | Bone | | 1880 | | | 44 | 0 | 81 | 30 | | --- | --- | --- | --- | | | - | |  | | |  | |  | | |  | | | X | |
| 113 | R168 | SPM | 1164 | Bone | | 1880 | | | 44 | 0 | 81 | 30 | | --- | --- | --- | --- | | | - | |  | | |  | |  | | |  | | | X | |
| 114 | R170 | SPM | 9421 | Bone | | 1908 | | | 37 | 30 | 100 | 0 | | --- | --- | --- | --- | | | IN4 | | Hap20 | | | EU818861 | | D | | | EU818893 | | |  | |
| 115 | R171 | SPM | 9409 | Bone | | 1913 | | | 38 | 0 | 72 | 0 | | --- | --- | --- | --- | | | IN1 | | Hap1 | | |  | | A | | |  | | | X | |
| 116 | R172 | SPM | 9412 | Bone | | 1913 | | | 38 | 0 | 74 | 0 | | --- | --- | --- | --- | | | IN3 | | Hap16 | | | EU818857 | | B | | | EU818891 | | | X | |
| 117 | R173 | SPM | 7702 | Bone | | 1900 | | | 37 | 30 | 98 | 0 | | --- | --- | --- | --- | | | - | |  | | |  | |  | | |  | | | X | |
| 118 | R174 | SPM | 9408 | Bone | | 1913 | | | 38 | 30 | 72 | 0 | | --- | --- | --- | --- | | | IN3 | | Hap15 | | | EU818856 | |  | | |  | | | X | |
| 119 | R176 | SPM | 20371 | Bone | | 1932 | | | 38 | 30 | 72 | 0 | | --- | --- | --- | --- | | | IN4 | | Hap21 | | | EU818862 | |  | | |  | | | X | |
| 120 | R178 | NM | 25572 | Bone | | 1981 | | | 51 | 47 | 87 | 13 | | --- | --- | --- | --- | | | IN5 | | Hap27 | | | EU818868 | | A | | |  | | | X | |
| 121 | R179 | NM | 10890 | Bone | | 1978 | | | 55 | 11 | 83 | 60 | | --- | --- | --- | --- | | | IN5 | | Hap22 | | |  | |  | | |  | | | X | |
| 122 | R180 | NM | 10889 | Bone | | 1976 | | | 54 | 28 | 89 | 58 | | --- | --- | --- | --- | | | IN1 | | Hap1 | | |  | |  | | |  | | | X | |
| 123 | R184 | NM | 10888 | Bone | | 1977 | | | 54 | 28 | 89 | 58 | | --- | --- | --- | --- | | | IN5 | | Hap22 | | |  | |  | | |  | | | X | |
| 124 | R185 | NM | 4540 | Bone | | 1959 | | | 55 | 11 | 80 | 19 | | --- | --- | --- | --- | | | IN5 | | Hap22 | | |  | |  | | |  | | | X | |
| 125 | R186 | NM | 4562 | Bone | | 1959 | | | 55 | 11 | 80 | 19 | | --- | --- | --- | --- | | | IN5 | | Hap28 | | | EU818869 | |  | | |  | | |  | |
| 126 | R187 | NM | 10505 | Bone | | 1976 | | | 53 | 26 | 85 | 26 | | --- | --- | --- | --- | | | IN10 | | Hap46 | | | EU818887 | |  | | |  | | |  | |
| 127 | R190 | NM | 1611 | Bone | | 1952 | | | 51 | 47 | 87 | 13 | | --- | --- | --- | --- | | | IN5 | | Hap22 | | |  | |  | | |  | | |  | |
| 128 | R191 | NM | 7180 | Bone | | 1942 | | | 51 | 47 | 87 | 13 | | --- | --- | --- | --- | | | IN5 | | Hap22 | | |  | |  | | |  | | |  | |
| 129 | R192 | NM | 32826 | Bone | | 1984 | | | 55 | 16 | 79 | 42 | | --- | --- | --- | --- | | | IN5 | | Hap22 | | |  | |  | | |  | | | X | |
| 130 | R193 | NM | 32825 | Bone | | 1984 | | | 55 | 16 | 79 | 42 | | --- | --- | --- | --- | | | IN5 | | Hap22 | | |  | |  | | |  | | | X | |
| 131 | R195 | NM | 2994 | Bone | | 1959 | | | 43 | 20 | 132 | 21 | | --- | --- | --- | --- | | | IN5 | | Hap22 | | |  | |  | | |  | | | X | |
| 132 | BM2 | BM | 3593 | Tooth | | NA | | | 60 | 0 | 28 | 0 | | --- | --- | --- | --- | | | IN8 | | Hap35 | | |  | |  | | |  | | |  | |
| 133 | BM4 | BM | 3985 | Tooth | | 1924 | | | 63 | 0 | 10 | 0 | | --- | --- | --- | --- | | | IN5 | | Hap22 | | |  | |  | | |  | | |  | |
| 134 | BM5 | BM | 3058 | Tooth | | 1919 | | | 63 | 0 | 10 | 0 | | --- | --- | --- | --- | | | IN5 | | Hap22 | | |  | |  | | |  | | | X | |
| 135 | BM7 | BM | 3057 | Tooth | | 1919 | | | 63 | 0 | 10 | 0 | | --- | --- | --- | --- | | | IN5 | | Hap22 | | |  | |  | | |  | | | X | |
| 136 | BM10 | BM | 2222 | Tooth | | 1922 | | | 63 | 0 | 10 | 0 | | --- | --- | --- | --- | | | IN5 | | Hap22 | | |  | |  | | |  | | |  | |
| 137 | BM11 | BM | 3826 | Tooth | | 1912 | | | 63 | 0 | 10 | 0 | | --- | --- | --- | --- | | | IN5 | | Hap22 | | |  | |  | | |  | | |  | |
| 138 | CZ13 |  |  | Muscle | | NA | | | 50 | 0 | 15 | 0 | | --- | --- | --- | --- | | | IN9 | | Hap43 | | |  | |  | | |  | | | X | |
| 139 | CZ14 |  |  | Muscle | | 1994 | | | 50 | 0 | 15 | 0 | | --- | --- | --- | --- | | | IN9 | | Hap43 | | |  | | A | | |  | | |  | |
| 140 | CZ15 |  |  | Muscle | | 2001 | | | 50 | 0 | 15 | 0 | | --- | --- | --- | --- | | | IN9 | | Hap43 | | |  | | A | | |  | | |  | |
| 141 | CZ16 |  |  | Muscle | | 2001 | | | 50 | 0 | 15 | 0 | | --- | --- | --- | --- | | | IN9 | | Hap43 | | |  | | A | | |  | | | X | |
| 142 | KZ3 | AZ | Char | Hair | | 2000 | | | 48 | 0 | 82 | 0 | | --- | --- | --- | --- | | | IN5 | | Hap22 | | |  | |  | | |  | | |  | |
| 143 | KZ4 | AZ | Maryan | Hair | | 2000 | | | 48 | 0 | 82 | 0 | | --- | --- | --- | --- | | | IN1 | | Hap1 | | |  | |  | | |  | | |  | |
| 144 | KZ5 | AZ | Tosca | Hair | | 2000 | | | 48 | 0 | 82 | 0 | | --- | --- | --- | --- | | | IN1 | | Hap1 | | |  | |  | | |  | | |  | |
| 145 | SCA | GB | AY034813 (700bp) | | |  | | | 63 | 0 | 10 | 0 | | --- | --- | --- | --- | | | =IN5 | |  | |  | | | |  | | | |  | | |
| 146 | BA1 | GB | AY034816 (700bp) | | |  | | | 57 | 0 | 25 | 0 | | --- | --- | --- | --- | | | =IN9 | |  | |  | | | |  | | | |  | | |
| 147 | BA2 | GB | AY034815 (700bp) | | |  | | | 57 | 0 | 25 | 0 | | --- | --- | --- | --- | | | =IN13 | |  | |  | | | |  | | | |  | | |
| 148 | BA3 | GB | AY03484 (700bp) | | |  | | | 57 | 0 | 25 | 0 | | --- | --- | --- | --- | | | =IN8 | |  | |  | | | |  | | | |  | | |
|  |  |  |  |  | |  | |  | |  | | 137 | | |  | | 56 | | |  | | | 104 | |

Sources: MM = Moscow Museum, SBM = St. Petersburg Museum, BM = Bergen Museum, AZ = Almaty Zoo, GB = GenBank.
